# Foreign Summary

**Published:** 1840-05-30

**Authors:** 


### FOREIGN SUMMARY.

*Apoplexy of the Eyes.* By Dr. Holscher.—A robust country girl, of eighteen, was hard at work in the harvest field one hot day; and had been frequently stooping to bind up sheaves, when, without any external cause, but with great congestion of blood about the head, she became suddenly blind. When examined a short time after, all sight was lost; the eyes were fixed and felt tense; the conjunctiva was very vascular, there was considerable congestion about the head, and the carotid arteries pulsated strongly. The pupils were widely dilated, and did not in the least contract even when a strong light was brought close to the eye. In the right eye, on viewing it from the side, a slight red tinge of the aqueous humour was perceptible; and with a lens a very small coagulum of blood was discovered lying at the bottom of the anterior chamber. The patient, except for her blindness, was in the same robust health which she had always enjoyed.

Feeling no doubt that the sudden loss of sight was produced by an equally sudden congestion of the eye by blood rushing, as in apoplexy, into the vessels of the choroid and retina, and producing paralysis of the latter, the author ordered a bleeding, to sixteen ounces, from the arm, cold lotions to the head, a saline foot-bath, saline medicines, and an extremely low diet. By a continuance of similar means, with the addition of mercury and a permanent blister at the back of the neck, the patient was so far recovered on the fourth day as to be able to distinguish objects placed before the eyes, and in three weeks she returned to the country with her sight perfectly restored.—*Brit. and For. Med. Rev.*, *from Hannoversche Annalen.*

